# The genome sequence of black elder, *Sambucus nigra* Linnaeus, 1753 (Adoxaceae)

**DOI:** 10.12688/wellcomeopenres.23147.1

**Published:** 2024-10-17

**Authors:** Maarten J. M. Christenhusz, Ilia J. Leitch, Royal Botanic Gardens Kew Genome Acquisition Lab, Darwin Tree of Life Barcoding collective, Plant Genome Sizing collective, Wellcome Sanger Institute Tree of Life Management, Samples and Laboratory team, Wellcome Sanger Institute Scientific Operations: Sequencing Operations, Wellcome Sanger Institute Tree of Life Core Informatics team, Tree of Life Core Informatics collective, Darwin Tree of Life Consortium

**Affiliations:** 1 Royal Botanic Gardens Kew, Richmond, England, UK; 2 Curtin University, Perth, Western Australia, Australia

**Keywords:** Sambucus nigra, European elder, genome sequence, chromosomal, Dipsacales

## Abstract

We present a genome assembly from an individual *Sambucus nigra* (the European elder; Streptophyta; Magnoliopsida; Dipsacales; Adoxaceae). The genome sequence has a total length of 11,813.70 megabases. Most of the assembly is scaffolded into 18 chromosomal pseudomolecules. The mitochondrial and plastid genome assemblies have lengths of 724.11 kilobases and 158.06 kilobases, respectively.

## Species taxonomy

Eukaryota; Viridiplantae; Streptophyta; Streptophytina; Embryophyta; Tracheophyta; Euphyllophyta; Spermatophyta; Magnoliopsida; Mesangiospermae; eudicotyledons; Gunneridae; Pentapetalae; asterids; campanulids; Dipsacales; Adoxaceae; *Sambucus*; *Sambucus nigra* L. (NCBI:txid4202).

## Background

Black elder, *Sambucus nigra* L., also known as elderberry or European elder, is a deciduous shrub or small tree up to 6, rarely 10 m tall. Together with the genera *Adoxa* (3 spp.) and *Viburnum* (about. 210 spp.), *Sambucus* (about 10 spp.) belongs to the elder family, Adoxaceae, although it is sometimes called Viburnaceae, Tinaceae or Sambucaceae, a nomenclatural conundrum ( Christenhusz *et al.*, 2017; Reveal, 2008).

Elder has grey bark and hollow young twigs. Its leaves are slightly foetid when crushed, but its bisexual flowers, which are formed into large terminal, umbel-like panicles, are highly fragrant. The flowers are visited by a large variety of flying insects, and after pollination are followed by black berries ( Atkinson & Atkinson, 2002) .

*Sambucus nigra* is found throughout Europe, north to Shetland and south-west to the Azores and east to the Caucasus ( POWO, 2024). It is closely related to the North American *S. canadensis* L., *S. cerulea* Raf. and *S. peruviana* Kunth, and the Macaronesian S *. palmensis* Link, with which it forms a species complex ( Applequist, 2015). It is widely cultivated and naturalised outside its native range, complicating the species delimitations in this complex.

In Britain and Ireland *S. nigra* is found on fertile soils up to about 500 m elevation in a wide range of habitats ranging from woodland and grassland to waste ground and roadsides. Studies have shown that it is resistant to grazing and hence often occurs around rabbit warrens ( Atkinson & Atkinson, 2002; Stroh *et al.*, 2020).

Black elder is associated with the Germanic legend of Mother Hulda, immortalised in the Brothers Grimm tales ( Grimm & Grimm, 1884). According to ancient legend she lived in an elder tree and therefore these trees were highly valued to fend off evil spirits. They were frequently planted near a well to secure safe water ( De Cleene & Lejeune, 2000).

The plant has been used by humans for centuries in a diversity of ways. For example, the scented flowers are frequently made into cordials or used to flavour liqueurs and other drinks or eaten in baked goods, pancakes or confectionary ( Christenhusz *et al.*, 2017). From a medicinal/pharmaceutical perspective, elderflower water has been used as a skin and eye tonic and berries are sometimes made into jams and syrup as a cold remedy, perhaps due to their high concentration of nutrients and antioxidants ( Młynarczyk *et al.*, 2018). However, both the berries and flowers can contain high concentration of cyanogenic glycosides (particularly when unripe), especially sambunigrin and prunasin. These are potentially life threatening as they can be hydrolysed leading to the release of cyanide ( Bromley *et al.*, 2005). Cooking it before consumption has therefore been recommended as heat has been shown to degrade the cyanogenic glycosides ( Młynarczyk *et al.*, 2018).

Cytological information about the species is limited to two chromosome counts from material sourced from the British Isles. Both studies report 2 *n* = 36, which agree with counts from other countries that, in addition, report that the karyotype comprises five metacentric, five submetacentric, six acrocentric and two telocentric chromosome pairs ( Benko-Iseppon & Morawetz, 1993; Ourecky, 1970).

*Sambucus* belongs to the family Adoxaceae which is a small family in the order Dipsacales, comprising just three genera ( *Sambucus, Adoxa* and *Viburnum*) and c. 150 to 200 species ( POWO, 2024). Since there are currently no other chromosome level whole genome assemblies for any species in Adoxaceae, the release of this genome provides the first deep genomic insights not only in *Sambucus nigra*, but also to the family as a whole.

## Genome sequence report

**Figure 1.**
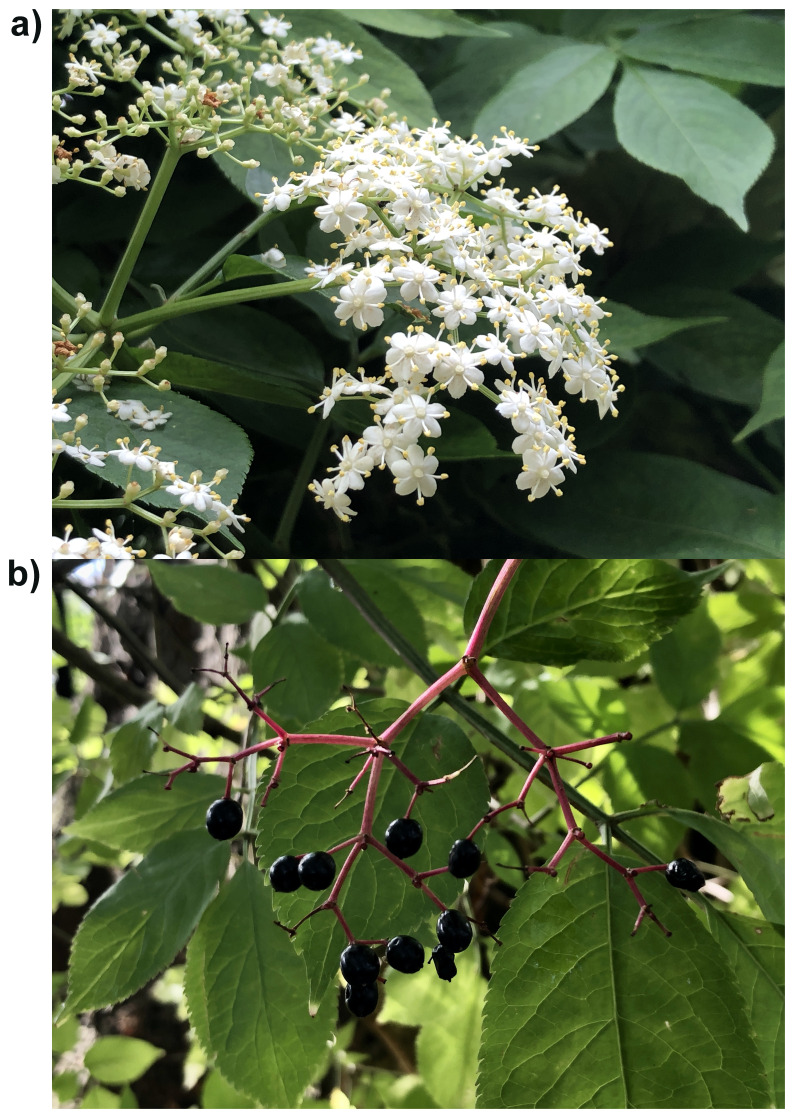
Photographs of the *Sambucus nigra* (daSamNigr1) specimen used for genome sequencing. **a**) in flower, **b**) in fruit.

The genome of a *Sambucus nigra* specimen ( Figure 1) was sequenced using Pacific Biosciences single-molecule HiFi long reads, generating a total of 329.92 Gb (gigabases) from 34.80 million reads, providing approximately 26-fold coverage. Using flow cytometry, the genome size (1C-value) was estimated to be 13.51 pg, equivalent to 13,210 Mb. Primary assembly contigs were scaffolded with chromosome conformation Hi-C data, which produced 954.04 Gb from 6,318.16 million reads, yielding an approximate coverage of 81-fold. Specimen and sequencing information is summarised in Table 1.

**Table 1.** Specimen and sequencing data for *Sambucus nigra*.

Project information			
**Study title**	Sambucus nigra (European elder)		
**Umbrella BioProject**	PRJEB56128		
**Species**	*Sambucus nigra*		
**BioSample**	SAMEA7522178		
**NCBI taxonomy ID**	4202		
Specimen information			
**Technology**	**ToLID**	**BioSample ** **accession**	**Organism part**
**PacBio long read sequencing**	daSamNigr1	SAMEA7522258	Leaf
**Hi-C sequencing**	daSamNigr1	SAMEA7522253	Leaf
**RNA sequencing**	daSamNigr1	SAMEA7522253	Leaf
Sequencing information			
**Platform**	**Run accession**	**Read count**	**Base count (Gb)**
**Hi-C Illumina NovaSeq 6000**	ERR10297858	6.32e+09	954.04
**PacBio Sequel IIe**	ERR10287560	8.52e+05	9.38
**PacBio Sequel IIe**	ERR10287562	1.24e+06	13.4
**PacBio Sequel IIe**	ERR10287567	8.71e+05	10.95
**PacBio Sequel IIe**	ERR10287568	8.42e+05	9.31
**PacBio Sequel IIe**	ERR10287571	4.40e+05	3.6
**PacBio Sequel IIe**	ERR10287577	4.56e+05	4.96
**PacBio Sequel IIe**	ERR10287578	2.16e+06	19.96
**PacBio Sequel IIe**	ERR10378061	2.26e+06	23.63
**PacBio Sequel IIe**	ERR10385536	2.23e+06	22.22
**PacBio Sequel IIe**	ERR10287565	1.88e+06	15.43
**PacBio Sequel IIe**	ERR10287566	1.86e+06	15.05
**PacBio Sequel IIe**	ERR10287569	1.37e+06	15.73
**PacBio Sequel IIe**	ERR10287572	1.83e+06	12.91
**PacBio Sequel IIe**	ERR10378060	2.14e+06	21.65
**PacBio Sequel IIe**	ERR10378062	2.24e+06	22.76
**PacBio Sequel IIe**	ERR10287561	1.95e+06	17.83
**PacBio Sequel IIe**	ERR10287563	1.87e+06	15.06
**PacBio Sequel IIe**	ERR10287564	1.88e+06	15.13
**PacBio Sequel IIe**	ERR10287570	1.05e+06	11.26
**PacBio Sequel IIe**	ERR10287573	1.08e+06	11.52
**PacBio Sequel IIe**	ERR10287574	9.16e+05	11.67
**PacBio Sequel IIe**	ERR10287575	1.03e+06	8.82
**PacBio Sequel IIe**	ERR10287576	2.35e+06	17.68
**RNA Illumina NovaSeq 6000**	ERR10378035	4.79e+07	7.23

**Figure 2.**
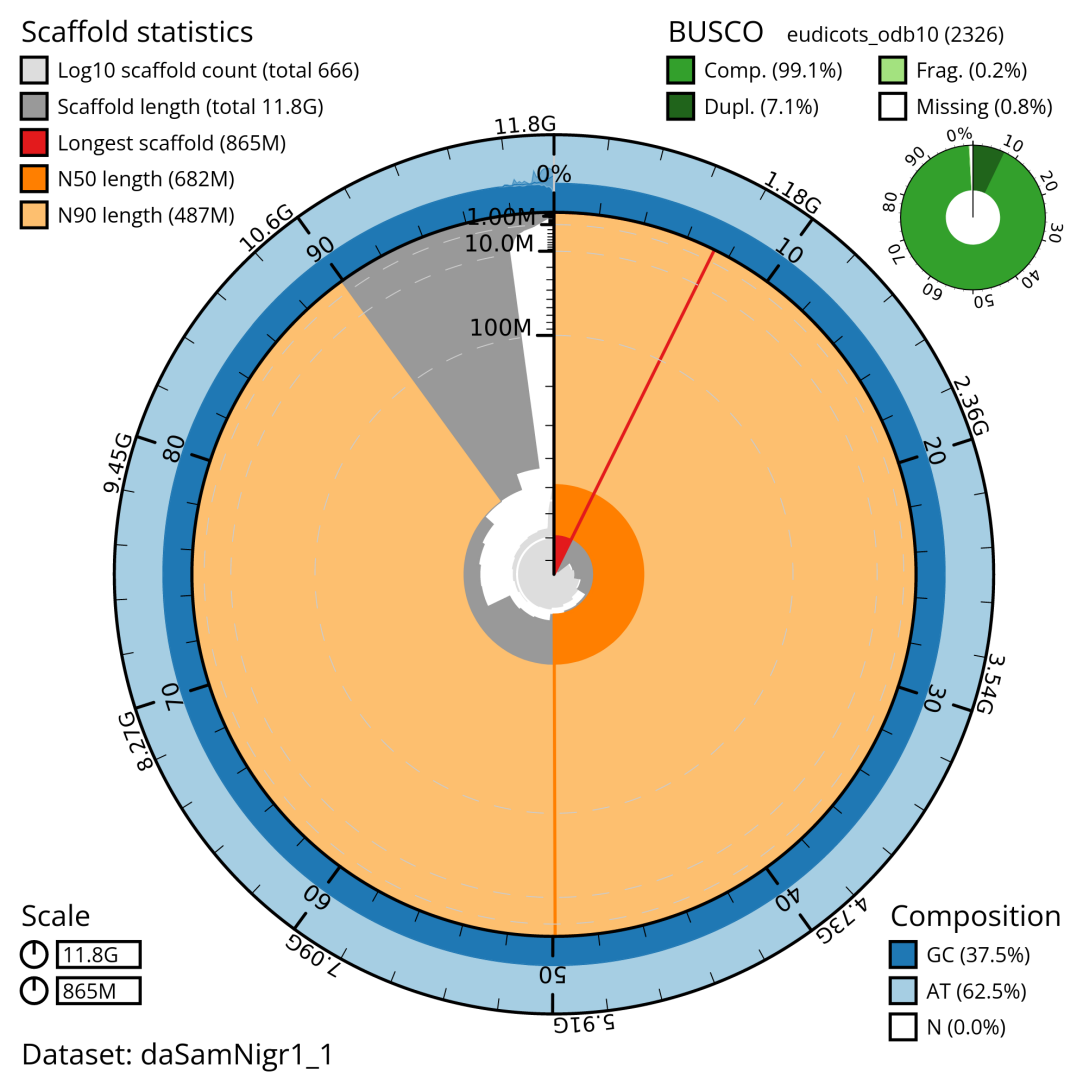
Genome assembly of *Sambucus nigra*, daSamNigr1.1: metrics. The BlobToolKit snail plot shows N50 metrics and BUSCO gene completeness. The main plot is divided into 1,000 size-ordered bins around the circumference with each bin representing 0.1% of the 11,814,595,023 bp assembly. The distribution of scaffold lengths is shown in dark grey with the plot radius scaled to the longest scaffold present in the assembly (865,431,811 bp, shown in red). Orange and pale-orange arcs show the N50 and N90 scaffold lengths (681,539,918 and 487,455,108 bp), respectively. The pale grey spiral shows the cumulative scaffold count on a log scale with white scale lines showing successive orders of magnitude. The blue and pale-blue area around the outside of the plot shows the distribution of GC, AT and N percentages in the same bins as the inner plot. A summary of complete, fragmented, duplicated and missing BUSCO genes in the eudicots_odb10 set is shown in the top right. An interactive version of this figure is available at https://blobtoolkit.genomehubs.org/view/daSamNigr1_1/dataset/daSamNigr1_1/snail.

**Figure 3.**
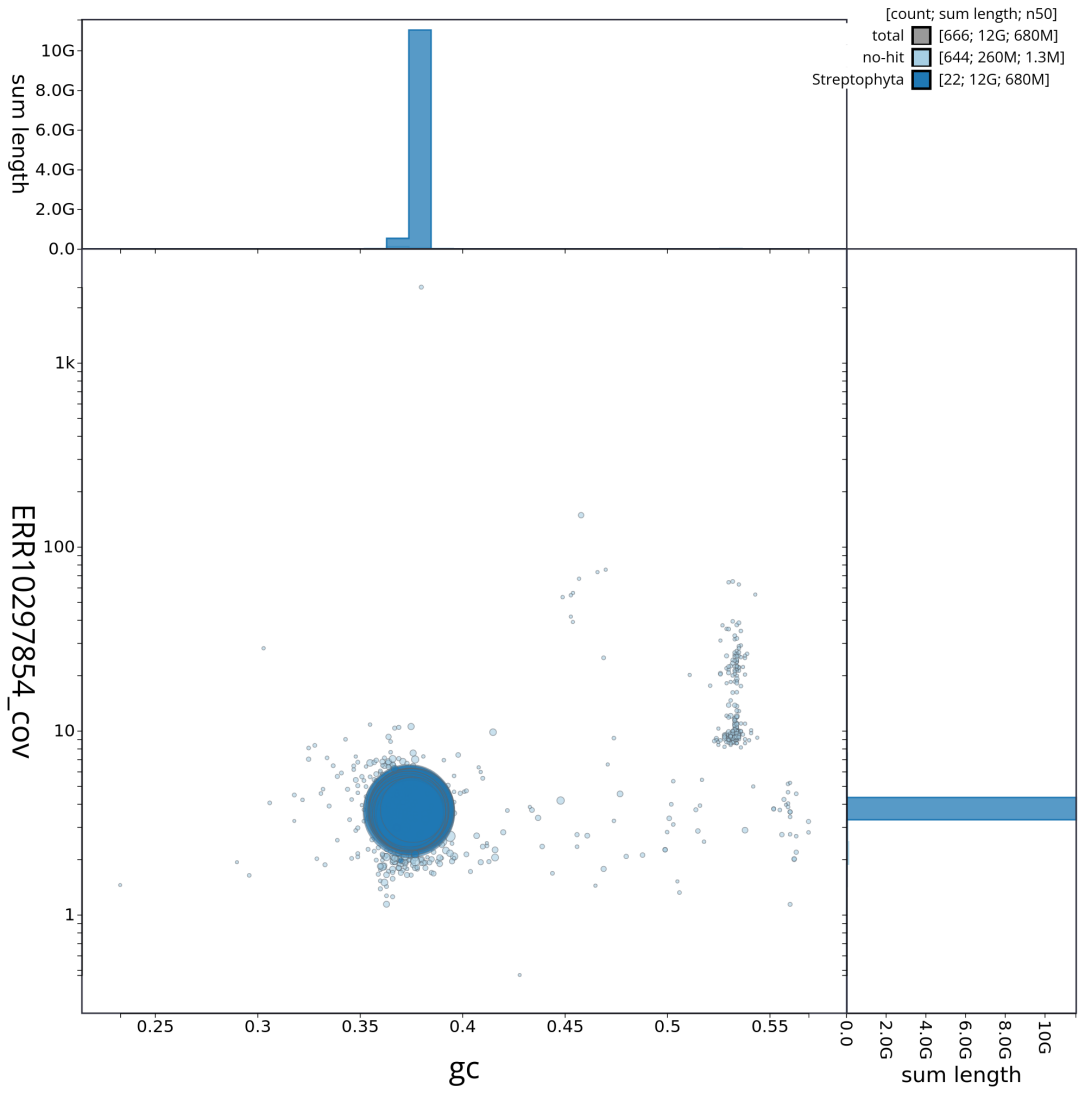
Genome assembly of *Sambucus nigra*, daSamNigr1.1: BlobToolKit GC-coverage plot. Sequences are coloured by phylum. Circles are sized in proportion to sequence length. Histograms show the distribution of sequence length sum along each axis. An interactive version of this figure is available at https://blobtoolkit.genomehubs.org/view/daSamNigr1_1/dataset/daSamNigr1_1/blob.

**Figure 4.**
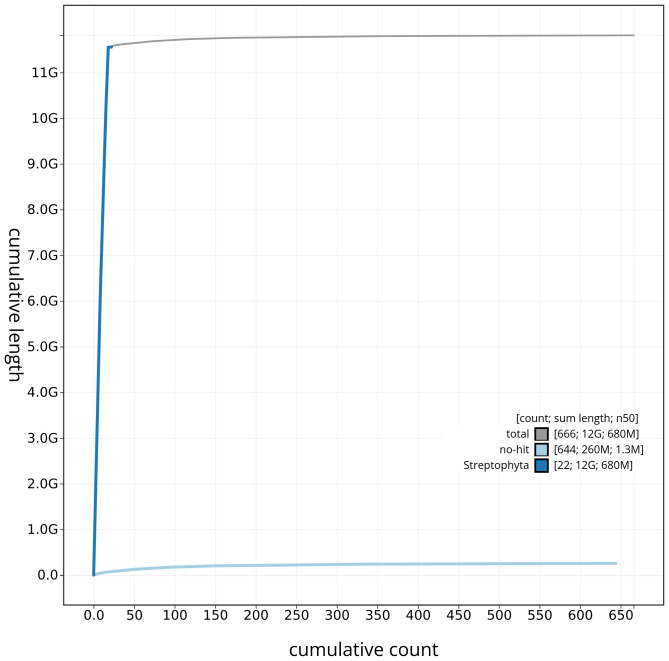
Genome assembly of *Sambucus nigra* daSamNigr1.1: BlobToolKit cumulative sequence plot. The grey line shows cumulative length for all sequences. Coloured lines show cumulative lengths of sequences assigned to each phylum using the buscogenes taxrule. An interactive version of this figure is available at https://blobtoolkit.genomehubs.org/view/daSamNigr1_1/dataset/daSamNigr1_1/cumulative.

**Figure 5.**
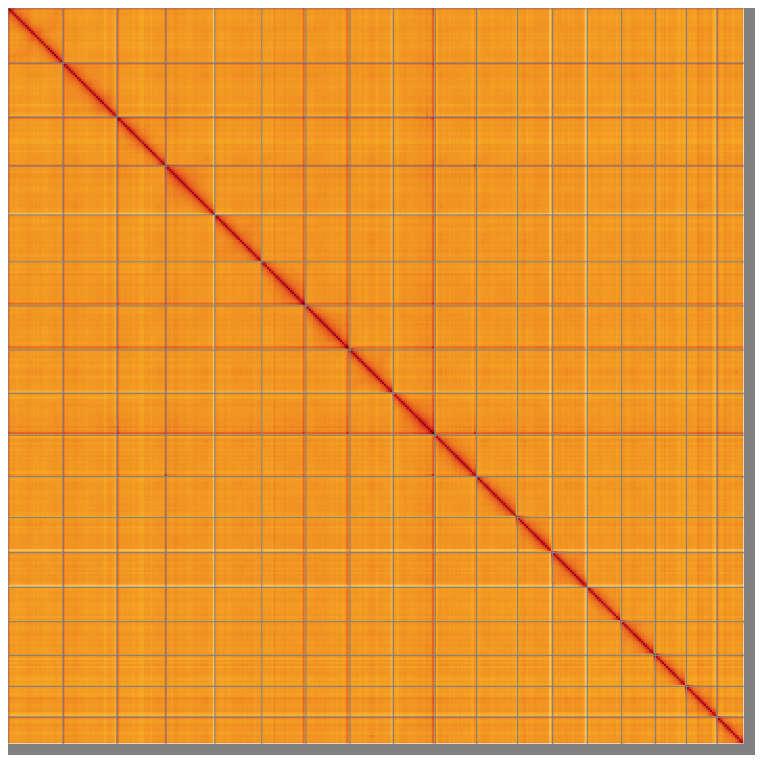
Genome assembly of *Sambucus nigra*, daSamNigr1.1: Hi-C contact map of the daSamNigr1.1 assembly, visualised using HiGlass. Chromosomes are shown in order of size from left to right and top to bottom. An interactive version of this figure may be viewed at https://genome-note-higlass.tol.sanger.ac.uk/l/?d=A8a95Fr3RJah-JHx7ESJDw.

Manual assembly curation corrected 152 missing joins or mis-joins and 32 haplotypic duplications, reducing the assembly length by 0.94%, and increasing the scaffold N50 by 4.62%. The final assembly has a total length of 11,813.70 Mb in 664 sequence scaffolds with a scaffold N50 of 681.5 Mb ( Table 2). The snail plot in Figure 2 provides a summary of the assembly statistics, while the distribution of assembly scaffolds on GC proportion and coverage is shown in Figure 3. The cumulative assembly plot in Figure 4 shows curves for subsets of scaffolds assigned to different phyla. Most (98.45%) of the assembly sequence was assigned to 18 chromosomal-level scaffolds. Chromosome-scale scaffolds confirmed by the Hi-C data are named in order of size ( Figure 5; Table 3). The following regions of this assembly are of undetermined order and orientation: Chromosome 3: 0–32.5 Mb, Chromosome 4: 0–20 Mb. While not fully phased, the assembly deposited is of one haplotype. Contigs corresponding to the second haplotype have also been deposited. The mitochondrial and plastid genomes were also assembled and can be found as contigs within the multifasta file of the genome submission.

**Table 2.** Genome assembly data for *Sambucus nigra*, daSamNigr1.1.

Genome assembly		
Assembly name	daSamNigr1.1	
Assembly accession	GCA_949130495.1	
*Accession of alternate * *haplotype*	*GCA_949130505.1*	
Span (Mb)	11,813.70	
Number of contigs	1,776	
Contig N50 length (Mb)	18.9	
Number of scaffolds	664	
Scaffold N50 length (Mb)	681.5	
Longest scaffold (Mb)	874.96	
Assembly metrics *		*Benchmark*
Consensus quality (QV)	61.1	*≥ 50*
*k*-mer completeness	100.0%	*≥ 95%*
BUSCO **	C:99.1%[S:91.9%,D:7.1%], F:0.2%,M:0.8%,n:2,326	*C ≥ 95%*
Percentage of assembly mapped to chromosomes	98.45%	*≥ 95%*
Organelles	Mitochondrial genome: 724.11 kb; plastid genome: 158.06 kb	*complete single* *alleles*

* Assembly metric benchmarks are adapted from column VGP-2020 of “Table 1: Proposed standards and metrics for defining genome assembly quality” from Rhie *et al.* (2021). ** BUSCO scores based on the eudicots_odb10 BUSCO set using version 5.4.3. C = complete [S = single copy, D = duplicated], F = fragmented, M = missing, n = number of orthologues in comparison. A full set of BUSCO scores is available at https://blobtoolkit.genomehubs.org/view/daSamNigr1_1/dataset/daSamNigr1_1/busco.

**Table 3.** Chromosomal pseudomolecules in the genome assembly of *Sambucus nigra*, daSamNigr1.

INSDC accession	Name	Length (Mb)	GC%
OX422207.1	1	865.43	37.5
OX422208.1	2	841.37	37.5
OX422209.1	3	772.39	37.5
OX422210.1	4	766.08	37.5
OX422211.1	5	735.9	37.5
OX422212.1	6	693.27	37.5
OX422213.1	7	690.06	37.5
OX422214.1	8	654.67	37.5
OX422215.1	9	681.54	37.5
OX422216.1	10	650.13	37.5
OX422217.1	11	643.74	37.5
OX422218.1	12	547.49	37.5
OX422219.1	13	545.35	37.5
OX422220.1	14	528.42	37.5
OX422221.1	15	538.51	37.5
OX422222.1	16	487.46	37.5
OX422223.1	17	484.16	37.5
OX422224.1	18	426.78	37.5
OX422225.1	MT	0.72	46.0
OX422226.1	Pltd	0.16	38.0

The estimated Quality Value (QV) of the final assembly is 61.1 with *k*-mer completeness of 100.0%, and the assembly has a BUSCO v5.4.3 completeness of 99.1% (single = 91.9%, duplicated = 7.1%), using the eudicots_odb10 reference set ( *n* = 2,326).

Metadata for specimens, BOLD barcode results, spectra estimates, sequencing runs, contaminants and pre-curation assembly statistics are given at https://links.tol.sanger.ac.uk/species/4202.

## Methods

### Sample acquisition, DNA barcoding and genome size estimation

The *Sambucus nigra* sample (specimen ID KDTOL10057, ToLID daSamNigr1) was hand-picked from the Royal Botanic Gardens, Kew (latitude 51.48, longitude –0.30) on 2020-08-26. The specimen was collected and identified by Maarten J. M. Christenhusz (Royal Botanic Gardens, Kew) and preserved by freezing at –80°C. The herbarium voucher associated with the sequenced plant (K001400695) has been deposited in the herbarium of Royal Botanic Gardens, Kew (K).

The initial species identification was verified by an additional DNA barcoding process according to the framework developed by Twyford *et al.* (2024). Part of the plant specimen was preserved in silica gel desiccant. DNA extracted from the dried plant was amplified by PCR for standard barcode markers, with the amplicons sequenced and compared to public sequence databases including GenBank and the Barcode of Life Database (BOLD). The barcode sequences for this specimen are available on BOLD ( Ratnasingham & Hebert, 2007). Following whole genome sequence generation, DNA barcodes were also used alongside the initial barcoding data for sample tracking through the genome production pipeline at the Wellcome Sanger Institute ( Twyford *et al.*, 2024). The standard operating procedures for the Darwin Tree of Life barcoding have been deposited on protocols.io ( Beasley *et al.*, 2023).

The genome size was estimated by flow cytometry using the fluorochrome propidium iodide and following the ‘one-step’ method as outlined in Pellicer *et al.* (2021). For this species, the General Purpose Buffer (GPB) supplemented with 3% PVP and 0.08% (v/v) beta-mercaptoethanol was used for isolation of nuclei ( Loureiro *et al.*, 2007), and the internal calibration standard was *Allium cepa* L. ‘Alice’ with an estimated 1C-value of 17,060 Mb ( Doležel *et al.*, 2007).

### Nucleic acid extraction

The workflow for high molecular weight (HMW) DNA extraction at the Wellcome Sanger Institute (WSI) Tree of Life Core Laboratory includes a sequence of core procedures: sample preparation and homogenisation, DNA extraction, fragmentation and purification. Detailed protocols are available on protocols.io ( Denton *et al.*, 2023). The daSamNigr1 sample was weighed and dissected on dry ice ( Jay *et al.*, 2023), and the leaf tissue was cryogenically disrupted using the Covaris cryoPREP ^®^ Automated Dry Pulverizer ( Narváez-Gómez *et al.*, 2023). HMW DNA was extracted using the Automated Plant MagAttract v1 protocol ( Sheerin *et al.*, 2023). HMW DNA was sheared into an average fragment size of 12–20 kb in a Megaruptor 3 system ( Todorovic *et al.*, 2023). Sheared DNA was purified by solid-phase reversible immobilisation, using AMPure PB beads to eliminate shorter fragments and concentrate the DNA ( Strickland *et al.*, 2023). The concentration of the sheared and purified DNA was assessed using a Nanodrop spectrophotometer and Qubit Fluorometer and Qubit dsDNA High Sensitivity Assay kit. Fragment size distribution was evaluated by running the sample on the FemtoPulse system.

RNA was extracted from leaf tissue of daSamNigr1 in the Tree of Life Laboratory at the WSI using the RNA Extraction: Automated MagMax™ *mir*Vana protocol ( do Amaral *et al.*, 2023). The RNA concentration was assessed using a Nanodrop spectrophotometer and a Qubit Fluorometer using the Qubit RNA Broad-Range Assay kit. Analysis of the integrity of the RNA was done using the Agilent RNA 6000 Pico Kit and Eukaryotic Total RNA assay.

### Hi-C preparation

Hi-C data were generated from daSamNigr1 leaf tissue, using the Arima-HiC v2 kit. Tissue was finely ground using cryoPREP, and then subjected to nuclei isolation using a modified protocol of the Qiagen QProteome Kit. After isolation, the nuclei were fixed, and the DNA crosslinked using a 37% formaldehyde solution. The crosslinked DNA was then digested using the restriction enzyme master mix. The 5’-overhangs were then filled in and labelled with biotinylated nucleotides and proximally ligated. An overnight incubation was carried out for enzymes to digest remaining proteins and for crosslinks to reverse. A clean up was performed with SPRIselect beads prior to library preparation. DNA concentration was quantified using the Qubit Fluorometer v2.0 and Qubit HS Assay Kit according to the manufacturer’s instructions.

### Library preparation and sequencing

Library preparation and sequencing were performed at the WSI Scientific Operations core. Pacific Biosciences HiFi circular consensus DNA sequencing libraries were constructed according to the manufacturers’ instructions. Libraries were prepared using the PacBio Express Template Preparation Kit v2.0 (Pacific Biosciences, California, USA) as per the manufacturer's instructions. The kit includes the reagents required for removal of single-strand overhangs, DNA damage repair, end repair/A-tailing, adapter ligation, and nuclease treatment. Library preparation also included a library purification step using 0.8X AMPure PB beads (Pacific Biosciences, California, USA) and size selection step to remove templates <5 kb using AMPure PB modified SPRI. DNA concentration was quantified using the Qubit Fluorometer v2.0 and Qubit HS Assay Kit and the final library fragment size analysis was carried out using the Agilent Femto Pulse Automated Pulsed Field CE Instrument and gDNA 55 kb BAC analysis kit. Samples were sequenced using the Sequel IIe system (Pacific Biosciences, California, USA). The concentration of the library loaded onto the Sequel IIe was within the manufacturer's recommended loading concentration range of 40–100 pM. The SMRT link software, a PacBio web-based end-to-end workflow manager, was used to set-up and monitor the run, as well as perform primary and secondary analysis of the data upon completion.

For Hi-C library preparation, DNA was fragmented to a size of 400 to 600 bp using a Covaris E220 sonicator. The DNA was then enriched, barcoded, and amplified using the NEBNext Ultra II DNA Library Prep Kit, following manufacturers’ instructions. The Hi-C sequencing was performed using paired-end sequencing with a read length of 150 bp on an Illumina NovaSeq 6000.

Poly(A) RNA-Seq libraries were constructed using the NEB Ultra II RNA Library Prep kit, following manufacturer’s instructions, and RNA sequencing was performed on the Illumina NovaSeq 6000 instrument.

### Genome assembly, curation and evaluation

***Assembly***

The HiFi reads were first assembled using Hifiasm ( Cheng *et al.*, 2021) with the --primary option. Haplotypic duplications were identified and removed using purge_dups ( Guan *et al.*, 2020). The Hi-C reads were mapped to the primary contigs using bwa-mem2 ( Vasimuddin *et al.*, 2019). The contigs were further scaffolded using the provided Hi-C data ( Rao *et al.*, 2014) in YaHS ( Zhou *et al.*, 2023) using the --break option. The scaffolded assemblies were evaluated using Gfastats ( Formenti *et al.*, 2022), BUSCO ( Manni *et al.*, 2021) and MERQURY.FK ( Rhie *et al.*, 2020). The organelle genomes were assembled using OATK ( Zhou, 2023).

***Curation***

The assembly was decontaminated using the Assembly Screen for Cobionts and Contaminants (ASCC) pipeline (article in preparation). Manual curation was primarily conducted using PretextView ( Harry, 2022), with additional insights provided by JBrowse2 ( Diesh *et al.*, 2023) and HiGlass ( Kerpedjiev *et al.*, 2018). Scaffolds were visually inspected and corrected as described by Howe *et al.* (2021). Any identified contamination, missed joins, and mis-joins were corrected, and duplicate sequences were tagged and removed. The process is documented at https://gitlab.com/wtsi-grit/rapid-curation (article in preparation).

***Evaluation of final assembly***

A Hi-C map for the final assembly was produced using bwa-mem2 ( Vasimuddin *et al.*, 2019) in the Cooler file format ( Abdennur & Mirny, 2020). To assess the assembly metrics, the *k*-mer completeness and QV consensus quality values were calculated in Merqury ( Rhie *et al.*, 2020). This work was done using the “sanger-tol/readmapping” ( Surana *et al.*, 2023a) and “sanger-tol/genomenote” ( Surana *et al.*, 2023b) pipelines. The genome evaluation pipelines were developed using nf-core tooling ( Ewels *et al.*, 2020) and MultiQC ( Ewels *et al.*, 2016), relying on the Conda package manager, the Bioconda initiative ( Grüning *et al.*, 2018), the Biocontainers infrastructure ( da Veiga Leprevost *et al.*, 2017), as well as the Docker ( Merkel, 2014) and Singularity ( Kurtzer *et al.*, 2017) containerisation solutions.

Table 4 contains a list of relevant software tool versions and sources.

**Table 4.** Software tools: versions and sources.

Software tool	Version	Source
BlobToolKit	4.2.1	https://github.com/blobtoolkit/blobtoolkit
BUSCO	5.3.2	https://gitlab.com/ezlab/busco
bwa-mem2	2.2.1	https://github.com/bwa-mem2/bwa-mem2
Cooler	0.8.11	https://github.com/open2c/cooler
Gfastats	1.3.6	https://github.com/vgl-hub/gfastats
Hifiasm	0.16.1-r375	https://github.com/chhylp123/hifiasm
HiGlass	1.11.6	https://github.com/higlass/higlass
Merqury	MerquryFK	https://github.com/thegenemyers/MERQURY.FK
OATK	0.1	https://github.com/c-zhou/oatk
PretextView	0.2	https://github.com/wtsi-hpag/PretextView
purge_dups	1.2.3	https://github.com/dfguan/purge_dups
sanger-tol/ genomenote	v1.0	https://github.com/sanger-tol/genomenote
sanger-tol/ readmapping	1.1.0	https://github.com/sanger-tol/readmapping/ tree/1.1.0
YaHS	yahs-1.1.91eebc2	https://github.com/c-zhou/yahs

### Wellcome Sanger Institute – Legal and Governance

The materials that have contributed to this genome note have been supplied by a Darwin Tree of Life Partner. The submission of materials by a Darwin Tree of Life Partner is subject to the **‘Darwin Tree of Life Project Sampling Code of Practice’**, which can be found in full on the Darwin Tree of Life website here. By agreeing with and signing up to the Sampling Code of Practice, the Darwin Tree of Life Partner agrees they will meet the legal and ethical requirements and standards set out within this document in respect of all samples acquired for, and supplied to, the Darwin Tree of Life Project.

Further, the Wellcome Sanger Institute employs a process whereby due diligence is carried out proportionate to the nature of the materials themselves, and the circumstances under which they have been/are to be collected and provided for use. The purpose of this is to address and mitigate any potential legal and/or ethical implications of receipt and use of the materials as part of the research project, and to ensure that in doing so we align with best practice wherever possible. The overarching areas of consideration are:

•   Ethical review of provenance and sourcing of the material

•   Legality of collection, transfer and use (national and international)

Each transfer of samples is further undertaken according to a Research Collaboration Agreement or Material Transfer Agreement entered into by the Darwin Tree of Life Partner, Genome Research Limited (operating as the Wellcome Sanger Institute), and in some circumstances other Darwin Tree of Life collaborators.

## Data Availability

European Nucleotide Archive: Sambucus nigra (European elder). Accession number PRJEB56128; https://identifiers.org/ena.embl/PRJEB56128 ( Wellcome Sanger Institute, 2023). The genome sequence is released openly for reuse. The *Sambucus nigra* genome sequencing initiative is part of the Darwin Tree of Life (DToL) project. All raw sequence data and the assembly have been deposited in INSDC databases. The genome will be annotated using available RNA-Seq data and presented through the Ensembl pipeline at the European Bioinformatics Institute. Raw data and assembly accession identifiers are reported in Table 1.

## Author information

Members of the Royal Botanic Gardens Kew Genome Acquisition Lab are listed here: https://doi.org/10.5281/zenodo.12625079.

Members of the Plant Genome Sizing collective are listed here: https://doi.org/10.5281/zenodo.7994306.

Members of the Darwin Tree of Life Barcoding collective are listed here: https://doi.org/10.5281/zenodo.12158331.

Members of the Wellcome Sanger Institute Tree of Life Management, Samples and Laboratory team are listed here: https://doi.org/10.5281/zenodo.12162482.

Members of Wellcome Sanger Institute Scientific Operations: Sequencing Operations are listed here: https://doi.org/10.5281/zenodo.12165051.

Members of the Wellcome Sanger Institute Tree of Life Core Informatics team are listed here: https://doi.org/10.5281/zenodo.12160324.

Members of the Tree of Life Core Informatics collective are listed here: https://doi.org/10.5281/zenodo.12205391.

Members of the Darwin Tree of Life Consortium are listed here: https://doi.org/10.5281/zenodo.4783558.
