# Supplementary material for: The genome sequence of black elder, Sambucus nigra Linnaeus, 1753 (Adoxaceae)
Source: Wellcome Open Res. 2024 Oct 17;9:609. [Version 1] doi: 10.12688/wellcomeopenres.23147.1 (PMC11555357; doi:10.12688/wellcomeopenres.23147.1)
Supplement: Peer review history

# Reviewer report: Reviewer response for version 1

**DOI:** 10.21956/wellcomeopenres.25490.r107305

**Published:** 2024-11-11

**Authors:** Sven Landrein

**Affiliations:** 1 Kadoorie Farm and Botanic Garden Corporation, Hong Kong, China

The denovo genome assembly (chromosome scale level) of *Sambucus nigra* is presented. The large genome of 11.8 Gb is matched by the depth of sequencing and the quality of the assembly is highly satisfactory. The bioinformatics tools for assembly and quality check are standard and although the results from these assemblies are briefly presented it seems not necessary for this report. As this is the first report of a denovo genome for Adoxaceae it is encouraging for future research in this family and the manuscript was well written. Some figures such as flow cytometry, organelle genomes are not shown. It is not clear why RNA was mentioned in the methods part as no transcriptome data is presented.

Are sufficient details of methods and materials provided to allow replication by others?

Yes

Is the rationale for creating the dataset(s) clearly described?

Yes

Are the datasets clearly presented in a useable and accessible format?

Yes

Are the protocols appropriate and is the work technically sound?

Yes

Reviewer Expertise:

Conservation genomics

I confirm that I have read this submission and believe that I have an appropriate level of expertise to confirm that it is of an acceptable scientific standard.

# Reviewer report: Reviewer response for version 1

**DOI:** 10.21956/wellcomeopenres.25490.r107303

**Published:** 2024-11-04

**Authors:** Chun-Lei Xiang

**Affiliations:** 1 Kunming Insitute of Botany, Kunming, Yunan, China

This study reported genome data of *Sambucu nigra* (Adoxaceae), providing the first deep genomic insights in the genus as well as the whole family. The paper is well written, figures are clear enough for the indexing. My only question is that the results of this article include genomes and organelles (such as mitochondria), so I suggest adding information on the treatment of organelles in the methods section.

Are sufficient details of methods and materials provided to allow replication by others?

Yes

Is the rationale for creating the dataset(s) clearly described?

Yes

Are the datasets clearly presented in a useable and accessible format?

Yes

Are the protocols appropriate and is the work technically sound?

Yes

Reviewer Expertise:

Taxonomy, Molecular Phylogeny

I confirm that I have read this submission and believe that I have an appropriate level of expertise to confirm that it is of an acceptable scientific standard.
